# The Effect of Parkinson's Disease on Patients Undergoing Lumbar Spine Surgery

**DOI:** 10.1155/2018/8428403

**Published:** 2018-06-27

**Authors:** Jeremy Steinberger, Jeffrey Gilligan, Branko Skovrlj, Christopher A. Sarkiss, Javier Z. Guzman, Samuel K. Cho, John M. Caridi

**Affiliations:** ^1^Department of Neurosurgery, Icahn School of Medicine at Mount Sinai, New York, NY, USA; ^2^Department of Neurosurgery, North Jersey Spine Group, Wayne, NJ, USA; ^3^Department of Orthopaedics, Icahn School of Medicine at Mount Sinai, New York, NY, USA

## Abstract

**Study Design:**

Retrospective Database Analysis.

**Objective:**

The purpose of this study was to assess characteristics and outcomes of patients with Parkinson's disease (PD) undergoing lumbar spine surgery for degenerative conditions.

**Methods:**

The Nationwide Inpatient Sample was examined from 2002 to 2011. Patients were included for study based on ICD-9-CM procedural codes for lumbar spine surgery and substratified to degenerative diagnoses. Incidence and baseline patient characteristics were determined. Multivariable analysis was performed to determine independent risk factors increasing incidence of lumbar fusion revision in PD patients.

**Results:**

PD patients account for 0.9% of all degenerative lumbar procedures. At baseline, PD patients are older (70.7 versus 58.9, *p* < 0.0001) and more likely to be male (58.6% male, *p* < 160.0001). Mean length of stay (LOS) was increased in PD patients undergoing lumbar fusion (5.1 days versus 4.0 days, *p* < 0.0001) and lumbar fusion revision (6.2 days versus 4.8 days, *p* < 180.0001). Costs were 7.9% (*p* < 0.0001) higher for lumbar fusion and 25.2% (*p* < 0.0001) higher for lumbar fusion revision in PD patients. Multivariable analysis indicates that osteoporosis, fluid/electrolyte disorders, blood loss anemia, and insurance status are significant independent predictors of lumbar fusion revision in patients with PD.

**Conclusion:**

PD patients undergoing lumbar surgery for degenerative conditions have increased LOS and costs when compared to patients without PD.

## 1. Introduction

Parkinson's disease (PD) is a neurodegenerative disorder characterized by resting tremors, rigidity, bradykinesia, postural instability, and gait disturbances [1]. The prevalence of PD in industrialized countries is estimated at 0.3% of the entire population with approximately 7 million people affected worldwide [2]. PD is an age-related disease which is rare before the age of 50, with a prevalence of about 1% in people over the age of 60 and up to 4% in people over the age of 80 [3, 4].

Apart from the neurodegenerative symptoms, patients with PD suffer from a wide variety of systemic and musculoskeletal dysfunctions. They are predisposed to falls due to a high incidence of visual impairment and autonomic dysfunction separate from neurodegenerative symptoms [5]. Epidemiologic studies suggest that approximately half of PD patients fall at least once as compared with a third of healthy ambulatory subjects greater than 60 years of age [6, 7]. These patients also suffer from osteoporosis, thus increasing their risk of bone fractures [8]. Musculoskeletal dysfunction in patients with PD leads to an increased incidence of muscle weakness and degenerative spondylarthroses resulting in scoliosis, thoracic kyphosis, and cervical deformity [9].

PD is increasingly recognized as an important cause of spinal disorders requiring surgical intervention [9]. However, spinal procedures can be complicated by underlying osteoporosis and severe musculoskeletal dysfunction in this population.

In this study, we investigate the effect of PD on patients undergoing lumbar spine surgery. The aim of this study is to identify the incidence, trend, risk factors, outcomes, and cost of lumbar spinal surgery for degenerative disease in PD patients.

## 2. Materials and Methods

The Nationwide Inpatient Sample (NIS) database, under the auspices of the Healthcare Cost and Utilization Project (HCUP) and administered by the Agency for Healthcare Research and Quality, was queried from 2002 to 2011 [10]. The NIS, which comprises a 20% stratified samples of all hospital discharges, is the largest all-payer hospital inpatient database in the US. This sample comprises approximately 8 million hospitalizations, and when sample weights are applied, it comprises approximately 40 million hospitalizations or 96% of all US hospital discharges each year. The NIS data contain patient demographics (e.g., race, age, and gender), hospital characteristics (e.g., teaching status, location, and size), and clinical outcomes (e.g., mortality, costs, and length of stay).

### 2.1. Sample Selection

PD was identified by the International Classification of Diseases, Ninth Revision, Clinical Modification (ICD-9-CM) code 332.0, which applies Parkinsonism characterized in the following forms: primary, idiopathic, or not otherwise specified. Patients were separated into two cohorts: patients with PD and those without PD. Hospitalizations were selected for the study based on ICD-9-CM procedural codes for lumbar spine procedures and further stratified to include only procedures for degenerative conditions of the lumbar spine. Only patients with hospitalizations that contained all of the demographics and clinical outcome measures were included. Since our search was conducted in this fashion, it is not known the amount of patients who had incomplete data that were excluded. The procedural codes used in this study are outlined in Table 1. Procedures were organized into three groups: lumbar fusion, lumbar fusion revision, and lumbar decompression without fusion.

### 2.2. Outcome Measures

Demographic data was analyzed, which included age, pay schedule, gender, race, modified Elixhauser Comorbidity Index, hospital characteristics, and surgical procedure. We chose the Elixhauser index for its ability to adjust for each single comorbidity's independent association with hospital death and its significant association with short- and long-term mortality as well as burden of diseases [11–13]. We have modified the Elixhauser index to exclude the point value of the neurological comorbidity as this includes the ICD-9-CM code for PD when utilizing the validated and updated comorbidity software provided by HCUP.

Perioperative complications were also chosen based on ICD-9-CM diagnosis codes (Supplementary Appendix A). We further analyzed hospitalization outcomes such as length of stay (LOS), costs, and mortality rates. All hospital charges were adjusted for inflation using the US Bureau of Labor statistics' yearly inflation calculator to represent charges in the year 2011 and converted into costs with the HCUP costs to charge ratio tool [14, 15].

### 2.3. Data Analysis

Statistical analysis was performed using SAS version 9.3 (SAS Institute, Cary, NC, USA). Chi-squared test was used for analysis of categorical variables and Student's *t*-test was used for continuous variables. Analysis took into account the complex survey design of the NIS and procedures such as *surveyfreq*, *surveymeans*, and *surveylogistic* being used for data analysis. Discharge weights, NIS stratum, and cluster (hospital identification) variables were included to correctly estimate variance and to produce national estimates from the stratified sample. Regression modeling for acute complications adjusting for PD, gender, race, hospital bed size, hospital region, and hospital location and modified Elixhauser index was performed to examine odds ratios for complications referencing PD patients to those without PD.

Multivariate analysis was performed to assess factors associated with lumbar fusion revision in the PD patient population. Factors included in multivariate analysis for lumbar fusion revision included the following: osteoporosis, age, race, gender, hospital size, region and location, insurance, and modified Elixhauser index. Two separate multivariate analyses not isolating the PD patient population but including all lumbar patients looked at the role PD +/− 6 osteoporosis had on lumbar fusion revision. These analyses included the same variables as the model isolating the PD patient population. Cochran–Armitage trend test was performed to assess PD trend of prevalence over time in patients undergoing degenerative lumbar spine surgery. Statistical significance was maintained at *p* < 0.05.

No Institutional Board Review approval was required for this study.

## 3. Results

**Figure 1:**
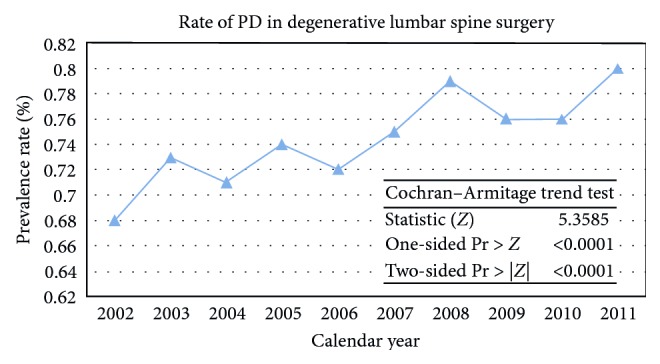
Stock plot demonstrating increased prevalence of Parkinson's disease patients undergoing lumbar spine surgery for degenerative conditions between the years 2002 and 2011.

A total of 19,211 PD patients underwent elective spine surgery from 2002 to 2011, with the prevalence significantly increasing over time (*p* < 0.0001) (Figure 1). Patients with PD were significantly older (70.7 versus 58.9, *p* < 0.0001) and more likely to be male (58.6% versus 47.1%, *p* < 0.0001) (Table 2). A greater proportion of PD patients had Medicare than non-PD patients (77.7% versus 41.1%, *p* < 0.0001) and more PD patients underwent lumbar decompression without fusion and lumbar fusion revision when compared to non-PD patients (*p* < 0.0001) (Table 2).

PD patients undergoing lumbar spine surgery had more comorbidities than those without PD (Table 3). Notably, patients with PD were more likely to have osteoporosis (7.9% versus 4.0%, *p* < 0.0001) and congestive heart failure (3.6% versus 1.9%, *p* < 0.0001). Despite the general pattern of greater comorbidities in patients with PD, this did not hold true for chronic pulmonary disease, rheumatoid arthritis, or obesity (Table 3).

Adjusted regression modeling for postoperative complications showed genitourinary complications' odds ratio (OR) = 1.5, confidence interval (CI) = 1.5–1.9, *p*=0.001) and postoperative hemorrhage (OR = 1.3, CI = 1.2–1.5, *p* ≤ 0.0001) as having significantly increased adjusted odds ratios amongst the complications analyzed (Table 4). As seen in Table 5, PD patients have significantly increased LOS with all procedures analyzed. When compared to non-PD patients, costs were significantly increased in lumbar fusion ($29,427 versus $27,272, *p* < 0.0001) and lumbar fusion revision ($39,885 versus $31,866, *p* < 0.0001). PD was not found to be associated with increased mortality in patients undergoing lumbar spine surgery (0.13% versus 0.11%, *p*=0.810).

Multivariate analysis performed on the PD patient population identified several independent factors that increase the odds of revision surgery (Table 6). Of note, PD patients with a diagnosis of osteoporosis (OR = 2.0, *p*=0.029) and Medicare (OR = 1.4, *p* < 0.0001) had increased likelihood of revision. Uninsured patients showed the most dramatic increased likelihood of revision surgery (OR = 9.84, *p* < 0.0001). Similar multivariate analysis on all patients undergoing degenerative lumbar spine surgery including PD patients also identified osteoporosis (OR = 1.3, CI = 1.2–1.4, *p* < 0.0001) as having increased odds of revision surgery (Supplementary Appendix B). However, a diagnosis of PD was not an independent risk factor for revision surgery. The combined diagnoses of PD and osteoporosis showed a significantly increased risk for lumbar fusion revision surgery (OR = 1.8, CI = 1.03–3.2, *p*=0.040) (Supplementary Appendix C).

## 4. Discussion

Adults older than 50 years are projected to be the fastest growing segment of the adult population. It is estimated that by 2050, a third of the American population will be over the age of 55 and 20% will be over 65 [16]. As such, a growing number of patients undergoing treatment for degenerative spinal conditions will have PD.

Despite its increasing disease burden, only six studies investigating spine surgery in the PD population exist presently [9, 17–21]. In this combined cohort of only 95 patients, complications were reported in 59% of all cases with 71% of patients achieving successful fusion following index surgery and 45% requiring revision surgery [22]. In patients who underwent decompression surgery alone, 100% required revision multilevel-instrumented fusion [22]. Satisfactory surgical outcome was noted in only 63% of patients [22].

Babat et al. [17] were the first to report on 14 patients with PD who underwent lumbar spine surgery and found an overall reoperation rate of 86% with hardware failure reported in 29% of patients. The authors opined that the primary mechanisms of failure were relentless kyphosis or segmental instability at the operated levels.

Kaspar et al. [19] assessed the postoperative complications in 24 PD patients undergoing all types of spinal surgery and reported a 21% revision rate, including 2 cases of pseudoarthrosis and 2 patients with recurrent stenosis. The authors concluded that symptoms and functional deficits of spinal disease were often masked by PD, which posed difficulties in diagnosis. However, in their series, the complication rates in PD patients were comparable to those in the general population, and it was the authors' opinion that spine symptoms improved concomitantly with successful surgery, unless the PD symptoms progressed or significant complications ensued.

Moon et al. [9] reported postoperative outcomes in twenty patients with PD undergoing lumbar fusion surgery for degenerative disease. In their series, only one patient (5%) had a satisfactory outcome. The average postoperative visual analog pain scale (VAS, 0 to 100 mm) was 55.2, whereas the mean preoperative VAS was 53.9. Radiological assessment showed successful fusion in 15 (75%) patients. The authors concluded that a poor surgical outcome might be inevitable due to the progressive natural history of PD and that surgical indications in patients with PD and spinal stenosis should be exercised with caution. It was the authors' opinion that even though implementing the proper surgical intervention is crucial in treating spinal disease in PD patients, the most important factor in the management of PD patients should be medical and/or surgical treatment of PD itself.

In the present study, a total of 19,211 patients with PD underwent elective lumbar spine surgery for degenerative diagnoses. There was an increasing national trend of PD patients undergoing lumbar spine surgery. The overall prevalence of PD is 1.6% in the population over age of 65 years 6 and 3.5% in those over age of 85 years [23] Additionally, PD patients were more often White males, which is in 7 accordance with the current epidemiologic data on PD [2, 24–26].

A larger proportion of patients with PD who underwent surgery had Medicare as their insurance (77.7 versus 41.1, *p* < 0.0001) compared to other patients, a finding which is not surprising given the older age of PD patients. Compared to non-PD patients, those with PD underwent a greater number of noninstrumented, lumbar decompression-only surgeries (55.4 versus 42.2, *p* < 0.0001). This could be explained by the increased age and greater number of comorbidities in PD patients, forcing surgeons to perform shorter, less complicated, and potentially safer surgeries on these patients.

Of all postoperative complications, only genitourinary (GU) and hemorrhagic complications were found to be significantly increased in the PD population. GU dysfunction predisposes to urinary retention and is one of the most common autonomic disorders in patients with PD, making this patient population increasingly vulnerable to postoperative GU complications [27]. Hypertension is a common perioperative problem in PD patients that has been associated with increased risk of intracerebral hemorrhage in patients undergoing deep brain stimulator implantation [28, 29]. The increased risk for hemorrhagic complications in our study suggest a need for tight perioperative pressure control with an increased role for surgical drains to decrease hemorrhagic complications in PD patients undergoing lumbar spine surgery.

Additionally, we found that PD patients undergoing lumbar spine surgery had greater LOS and hospitalization costs associated with fusion and fusion revision surgery but not lumbar decompression surgery. While a greater percentage of PD patients underwent decompression surgery without fusion, it is important to decipher whether those patients who undergo decompression surgery alone have higher incidence of revision surgery as has been the finding of previously published studies [22].

Multivariate analysis assessing risk factors associated with lumbar revision surgery in PD patients found that insurance status and osteoporosis were associated with revision following the index procedure. In terms of insurance, uninsured patients were found to have a significantly increased odds ratio of revision surgery (OR = 9.84, *p* < 0.0001). Uninsured patients, secondary to decreased access to healthcare, likely present with advanced PD, multiple untreated comorbidities, and more severe spinal pathology requiring complex procedures with higher failure rates. As was theorized by other authors on this topic, adequate medical and surgical control of PD is of great importance as it may significantly affect postoperative outcomes in patients undergoing spine surgery [9].

Multiple studies have confirmed the association between osteoporosis and PD [30, 31]. This is not only associated with increased age but also with disorders of bone metabolism [32]. This factor is especially aggravated in women, as most women with PD are also postmenopausal [18]. Studies have shown that low bone mineral density values in PD patients are closely correlated with disease severity, increased bone turnover, vitamin D deficiency, and poor nutritional status [33–35]. Low vitamin D levels are associated with increased fracture risk, poor musculoskeletal coordination, and poor muscle tone [9]. When taken together, these factors are important reasons for poor surgical outcomes in PD patients.

This study found that a diagnosis of PD was not found to be an independent risk factor for revision lumbar fusion surgery. However, a combined diagnosis of PD and osteoporosis was found to significantly increase the likelihood of lumbar fusion revision surgery. The poor bone quality in osteoporotic individuals together with the severe musculoskeletal dysfunction associated with PD appears to have significant negative effects on the likelihood of a positive spinal fusion outcome in PD patients, a finding which was also emphasized by Moon et al. [9].

## 5. Conclusion

This study was able to evaluate the characteristics and outcomes of patients with PD undergoing lumbar spine surgery for degenerative conditions using a large national database. PD patients had increased LOS and overall costs but did not have an increased risk of postoperative mortality. PD in itself was not found to be a risk factor for revision lumbar fusion surgery; however, PD and a diagnosis of osteoporosis significantly increased the likelihood of fusion revision. While the national trend in PD patient's undergoing elective lumbar surgery is rising, surgeons must be aware of the less favorable outcomes of lumbar spine surgery in patients with PD. PD patients should be treated with greater caution than the general population, and adequate medical and surgical control of PD prior to spine surgery may allow for improved diagnosis and better outcomes in this patient population. Though not investigated in this study, future research could inspect if the rate of readmissions and functional outcomes following surgical intervention differs in the PD patient population. As further data are collected to study the various complications associated with PD, more work can be done to establish strategies and protocols to reduce these complications and help optimize the care and outcomes of PD patients.

## Figures and Tables

**Table 1:** ICD-9-CM procedural and diagnosis codes used.

ICD-9-CM procedural and diagnosis codes	
Procedural codes	
81.04	Dorsal and dorsolumbar fusion, anterior technique
81.05	Dorsal or dorsolumbar fusion, posterior technique
81.06	Lumbar and lumbosacral fusion, anterior technique
81.07	Lumbar and lumbosacral fusion, lateral transverse process technique
81.08	Lumbar and lumbosacral fusion, posterior technique
81.34	Refusion of dorsal and dorsolumbar spine, anterior technique
81.35	Refusion of dorsal and dorsolumbar spine, posterior technique
81.36	Refusion of lumbar and lumbosacral spine, anterior technique
81.37	Refusion of lumbar and lumbosacral spine, lateral transverse process technique
81.38	Refusion of lumbar and lumbosacral spine, posterior technique
03.09	Posterior lumbar decompression without fusion

Diagnosis codes	
721.3	Lumbosacral spondylosis without myelopathy
721.42	Lumbar region, spondylogenic compression of lumbar spinal cord
722.1	Lumbar intervertebral disc without myelopathy
722.52	Lumbar or lumbosacral intervertebral disc
722.73	Lumbar region, intervertebral disc disorder with myelopathy
722.83	Lumbar region, postlaminectomy syndrome
722.93	Lumbar region, other and unspecified disc disorder
724.02	Lumbar region, spinal stenosis

ICD-9-CM: International Classification of Diseases, Ninth Edition, Clinical Modification.

**Table 2:** Patients with and without PD undergoing lumbar spine surgery.

Demographics	No PD	PD	*p* value
Mean age	58.9	70.7	<0.0001

Age groups			<0.0001
0–44	18.53	0.45	
45–64	42.09	21.57	
>65	39.31	77.94	

Gender			
Male	47.07	58.61	<0.0001
Female	52.93	41.39	<0.0001

Race			<0.0001
White	65.08	69.81	
Black	4.87	1.42	
Hispanic	4.17	3.75	
Asian	0.78	1.31	
Native American	0.29	0.15	
Other	1.75	1.62	
Missing	23.06	21.93	

Insurance			<0.0001
Medicare	41.13	77.66	
Medicaid	6.37	3.50	
Private	41.96	17.04	
Uninsured	0.83	0.34	
Other	9.52	1.35	
Missing	0.19	0.11	

Procedures			
Lumbar fusion	53.98	40.56	<0.0001
Lumbar decompression without fusion	42.23	55.42	<0.0001
Lumbar fusion revision	3.79	4.02	0.488

Modified Elixhauser index	0.32	0.70	<0.0001

PD: Parkinson's disease.

**Table 3:** Comorbidities of patients with and without PD.

Comorbidities	No PD	PD	*p* value
Congestive heart failure	1.94	3.61	<0.0001
Valvular heart disease	2.86	4.34	<0.0001
Pulmonary circulation disease	0.51	0.87	0.001
Peripheral vascular disease	2.43	3.12	0.006
Hypertension	49.50	55.53	<0.0001
Paralysis	1.65	2.73	<0.0001
Chronic pulmonary disease	13.99	10.62	<0.0001
Diabetes w/o chronic complications	14.88	14.22	0.276
Diabetes w/chronic complications	1.54	2.26	0.000
Hypothyroidism	9.56	12.99	<0.0001
Renal failure	1.68	2.39	0.001
Liver disease	0.76	0.33	0.002
Peptic ulcer disease	0.02	0.05	0.160
Acquired immune deficiency syndrome	0.03	0.05	0.544
Lymphoma	0.23	0.22	0.869
Metastatic cancer	0.16	0.16	0.916
Solid tumor w/o metastasis	0.35	0.76	<0.0001
Rheumatoid arthritis	2.82	2.61	0.438
Coagulopathy	1.21	2.16	<0.0001
Obesity	10.18	5.79	<0.0001
Weight loss	0.38	0.91	<0.0001
Fluid and electrolyte disorders	6.15	8.46	<0.0001
Chronic blood loss anemia	0.87	1.01	0.374
Deficiency anemia	6.89	8.69	<0.0001
Alcohol abuse	0.92	0.54	0.014
Drug abuse	0.79	0.50	0.041
Psychoses	1.69	2.79	<0.0001
Depression	10.94	12.46	0.002
Osteoporosis	3.95	7.89	<0.0001

PD: Parkinson's disease.

**Table 4:** Adjusted complication odds ratio in PD patients undergoing lumbar surgery^*∗*^.

Complications	Odds ratio	Low 95% CI	High 95% CI	*p* value
Cerebrovascular accident	0.49	0.07	3.58	0.484
Respiratory complication	0.85	0.56	1.29	0.444
Cardiac complication	1.01	0.72	1.43	0.940
Deep venous thrombosis	1.76	0.98	3.17	0.061
Peripheral vascular disease	1.38	0.45	4.31	0.575
Neurological complication	2.03	0.99	4.16	0.052
Genitourinary complication	1.47	1.16	1.87	0.001
Postoperative shock	1.24	0.40	3.87	0.716
Wound complication	0.88	0.12	6.47	0.904
Pulmonary embolism	<0.001	<0.001	<0.001	<0.0001
Postoperative infection	1.48	0.90	2.43	0.123
Postoperative hemorrhage	1.30	1.16	1.47	<0.0001
Postoperative pneumonia	0.39	0.05	2.85	0.354
Myocardial infarction	1.16	0.42	3.15	0.778
Arrhythmia	0.99	0.62	1.60	0.977
Death	0.53	0.17	1.667	0.277

PD: Parkinson's disease. ^*∗*^Regression modeling adjusting for gender, race, hospital (bed size, region, and location), and modified Elixhauser index—reference for PD patients without PD.

**Table 5:** Length of stay and costs for patients with PD undergoing degenerative lumbar spine surgery.

	Length of stay (LOS)			Costs ($USD)		
	No PD	PD	*p* value	No PD	PD	*p* value
Lumbar fusion	4.00	5.13	<0.0001	$27,272	$29,427	<0.0001
Lumbar decompression	3.28	4.15	<0.0001	$12,366	$12,469	0.717
Lumbar fusion revision	4.80	6.24	<0.0001	$31,866	$39,885	<0.0001

PD: Parkinson's disease.

**Table 6:** Multivariate analysis assessing risk factors associated with lumbar revision surgery in patients with PD.

Risk factor	Odds ratio	Low 95% CI	High 95% CI	*p* value
Osteoporosis	1.98	1.07	3.65	0.029
Black	0.26	0.03	2.52	<0.0001
Hispanic	0.94	0.33	2.67	<0.0001
Asian	<0.001	<0.001	<0.001	<0.0001
Native American	<0.001	<0.001	<0.001	<0.0001
Other	0.57	0.18	1.80	<0.0001
Female	1.07	0.72	1.58	0.750
Age	0.95	0.93	0.97	<0.0001
Small hospital	0.92	0.36	2.40	0.953
Medium hospital	0.81	0.49	1.33	0.591
Teaching hospital	1.40	0.92	2.14	0.118
Midwest	1.22	0.51	2.87	0.653
South	1.59	0.75	3.38	0.350
West	1.72	0.79	3.75	0.209
Urban hospital	1.02	0.43	2.42	0.964
Modified Elixhauser index	1.01	0.95	1.06	0.856
Medicare	1.37	0.80	2.35	<0.0001
Medicaid	0.92	0.38	2.23	<0.0001
Uninsured	9.84	1.31	73.87	<0.0001
Other	0.97	0.21	4.48	0.004
Missing	<0.001	<0.001	<0.001	<0.0001

Race reference: white; hospital reference: large hospital; region reference: northeast; insurance reference: private.

## Data Availability

The data used to support the findings of this study are available from the corresponding author upon request.

## Supplementary Materials

Supplementary Materials Perioperative complications were also chosen based on ICD-9-CM diagnosis codes which are shown in Appendix A. Appendix B demonstrates the multivariate analysis on all patients undergoing degenerative lumbar spine surgery, including PD. Appendix C demonstrates the logistic regression with combined diagnoses of PD with other risk factors.
