# Supplementary material for: The Effect of Parkinson's Disease on Patients Undergoing Lumbar Spine Surgery
Source: Parkinsons Dis. 2018 Jun 27;2018:8428403. doi: 10.1155/2018/8428403 (PMC6051025; doi:10.1155/2018/8428403)
Supplement: Supplementary Materials — Perioperative complications were also chosen based on ICD-9-CM diagnosis codes which are shown in Appendix A. Appendix B demonstrates the multivariate analysis on all patients undergoing degenerative lumbar spine surgery, including PD. Appendix C demonstrates the logistic regression with combined diagnoses of PD with other risk factors. [file 8428403.f1.zip › Appendix C (1)_PD_2346744.docx]

| Appendix C. Logistic Regression Assessing Risk Factors for Lumbar Fusion Revision in Patients Undergoing Degenerative Lumbar Spine Surgery | | | | |
| --- | --- | --- | --- | --- |
| Risk Factor | **Odds Ratio** | **Low 95% CI** | **High 95% CI** | ***p*-value** |
| PD + Osteoporosis | 1.82 | 1.03 | 3.22 | 0.040 |
| Black | 0.90 | 0.82 | 0.99 | 0.002 |
| Hispanic | 0.77 | 0.70 | 0.86 | 0.779 |
| Asian | 0.60 | 0.48 | 0.76 | 0.025 |
| Native American | 0.67 | 0.47 | 0.95 | 0.387 |
| Other | 0.70 | 0.59 | 0.82 | 0.269 |
| Female | 1.21 | 1.17 | 1.25 | <.0001 |
| Age | 0.99 | 0.99 | 0.99 | <.0001 |
| Small Hospital | 0.91 | 0.74 | 1.12 | 0.388 |
| Medium Hospital | 0.98 | 0.84 | 1.15 | 0.738 |
| Teaching Hospital | 1.22 | 1.05 | 1.41 | 0.011 |
| Midwest | 0.99 | 0.79 | 1.23 | 0.214 |
| South | 1.16 | 0.96 | 1.42 | 0.147 |
| Midwest | 1.15 | 0.95 | 1.39 | 0.195 |
| Urban Hospital | 1.68 | 1.19 | 2.39 | 0.004 |
| Modified Elixhauser Index | 1.00 | 0.99 | 1.00 | 0.407 |
| Medicare | 1.35 | 1.27 | 1.44 | <.0001 |
| Medicaid | 1.13 | 1.02 | 1.24 | 0.592 |
| Uninsured | 0.67 | 0.53 | 0.84 | <.0001 |
| Other | 1.33 | 1.23 | 1.43 | 0.000 |
| Missing | 1.28 | 0.84 | 1.94 | 0.384 |

PD: Parkinson’s disease; CI: Confidence interval
