# Supplementary material for: The Effect of Parkinson's Disease on Patients Undergoing Lumbar Spine Surgery
Source: Parkinsons Dis. 2018 Jun 27;2018:8428403. doi: 10.1155/2018/8428403 (PMC6051025; doi:10.1155/2018/8428403)
Supplement: Supplementary Materials — Perioperative complications were also chosen based on ICD-9-CM diagnosis codes which are shown in Appendix A. Appendix B demonstrates the multivariate analysis on all patients undergoing degenerative lumbar spine surgery, including PD. Appendix C demonstrates the logistic regression with combined diagnoses of PD with other risk factors. [file 8428403.f1.zip › appendix a (1)_PD_2346742.docx]

| Appendix A. *ICD-9-CM* Diagnosis Codes for Complications | |
| --- | --- |
| Acute Complication | **Codes** |
| Cerebrovascular | 997.02 |
| Respiratory | 997.3 |
| Cardiac | 997.1 |
| Deep Venous Thrombosis | 451.11, 453.4, 453.9, 451.19, 451.2, 451.81, 453.40, 453.41 |
| Peripheral Vascular | 997.2 |
| Nervous System | 997.00, 997.01, 997.0 |
| Genitourinary | 997.5 |
| Postoperative Shock | 998.0, 998.00, 998.01, 998.02, 998.09 |
| Pulmonary Embolism | 415.1, 415.11, 415.19 |
| Postoperative Infection | 998.59 |
| Acute post-operative hemorrhage | 285.1 |
| Postoperative Infection | 998.59 |
| Postoperative hemorrhage | 285.1 |
| Postoperative Pneumonia | 480.0, 480.1, 480.2, 480.3, 480.8, 487.0, 484.1, 484.2, 484.3, 484.4, 484.5, 484.6, 484.7, 484.8, 480.9, 481, 482.0, 482.1, 482.2, 482.3, 482.30, 482.31, 482.32, 482.39, 482.4, 482.41, 482.42, 482.49, 482.8, 482.81, 482.82, 48.283, 482.84, 482.89, 482.9, 483, 483.0, 483.1, 483.8 |
| Myocardial Infarction | 410, 410.01, 410.11, 410.2, 410.21, 410.3, 410.31, 410.4, 410.41, 410.5, 410, 410.6, 410.9, 410.91 |
| Arrhythmia | 427.0, 427.1, 427.2 |

ICD-9-CM: International classification of diseases, ninth revision, clinical modification
