# Supplementary material for: The Effect of Parkinson's Disease on Patients Undergoing Lumbar Spine Surgery
Source: Parkinsons Dis. 2018 Jun 27;2018:8428403. doi: 10.1155/2018/8428403 (PMC6051025; doi:10.1155/2018/8428403)
Supplement: Supplementary Materials — Perioperative complications were also chosen based on ICD-9-CM diagnosis codes which are shown in Appendix A. Appendix B demonstrates the multivariate analysis on all patients undergoing degenerative lumbar spine surgery, including PD. Appendix C demonstrates the logistic regression with combined diagnoses of PD with other risk factors. [file 8428403.f1.zip › Appendix B (1)_PD_2346743.docx]

| Appendix B. Multivariate of all Patients with Degenerative Lumbar Spine Diagnoses Showing Associated Factors Increasing Odds of Lumbar Fusion Revision | | | | |
| --- | --- | --- | --- | --- |
| Risk Factor | **Odds Ratio** | **Low 95% CI** | **High 95% CI** | ***p*-value** |
| PD | 1.19 | 0.97 | 1.44 | 0.093 |
| Osteoporosis | 1.30 | 1.18 | 1.44 | <0.0001 |
| Black | 0.90 | 0.83 | 0.99 | 0.002 |
| Hispanic | 0.77 | 0.69 | 0.86 | 0.791 |
| Asian | 0.60 | 0.47 | 0.75 | 0.021 |
| Native American | 0.67 | 0.47 | 0.95 | 0.397 |
| Other | 0.70 | 0.59 | 0.82 | 0.267 |
| Female | 1.19 | 1.15 | 1.23 | <0.0001 |
| Age | 0.99 | 0.99 | 0.99 | <0.0001 |
| Small Hospital | 0.91 | 0.74 | 1.12 | 0.391 |
| Medium Hospital | 0.98 | 0.84 | 1.15 | 0.741 |
| Teaching Hospital | 1.22 | 1.05 | 1.41 | 0.011 |
| Midwest | 0.99 | 0.80 | 1.23 | 0.213 |
| South | 1.17 | 0.96 | 1.42 | 0.143 |
| West | 1.15 | 0.95 | 1.40 | 0.193 |
| Urban Hospital | 1.68 | 1.19 | 2.39 | 0.003 |
| Modified Elixhauser Index | 1.00 | 0.99 | 1.00 | 0.359 |
| Medicare | 1.34 | 1.26 | 1.43 | <0.0001 |
| Medicaid | 1.13 | 1.02 | 1.24 | 0.583 |
| Uninsured | 0.67 | 0.53 | 0.84 | <0.0001 |
| Other | 1.32 | 1.22 | 1.43 | 0.000 |
| Missing | 1.27 | 0.84 | 1.93 | 0.390 |

PD: Parkinson’s disease
